# Transcript PHF19-207 May Be a Long Non-Coding RNA with Tumor-Promoting Role in Colon Cancer

**DOI:** 10.3390/biom15070957

**Published:** 2025-07-02

**Authors:** Dunja Pavlovic, Tamara Babic, Sofija Ignjatovic, Katarina Pavlovic, Sandra Dragicevic, Aleksandra Nikolic

**Affiliations:** Institute of Molecular Genetics and Genetic Engineering, University of Belgrade, Vojvode Stepe 444a, 11000 Belgrade, Serbia

**Keywords:** colon cancer, PHF19-207, alternative transcript, next-generation sequencing, diagnostic biomarker

## Abstract

Recent pan-cancer transcriptome analysis has revealed differential activity of two alternative *PHF19* gene promoters in malignant versus non-malignant gut mucosa. One of these promoters upregulated in colon cancer leads to the expression of the PHF19-207 transcript, suggesting its potential role in tumor promotion. The objective of this study was to investigate the function of PHF19-207 using in silico tools and publicly available data, as well as to assess its expression in colon cancer. Expression analyses were conducted via qPCR and RNA sequencing on RNA extracted from the immortalized colonic epithelial cell line HCEC-1CT, as well as a series of colon cancer cell lines cultured in both 2D and 3D environments. The expression of PHF19-207 was found to be elevated in all malignant cell lines compared to the non-malignant HCEC-1CT cell line in both culture conditions, with the most prominent increase observed in cell lines derived from advanced stages of the disease and in the HCEC-1CT cell line overexpressing KRAS. Furthermore, the PHF19-207 transcript was detected in exosomes derived from malignant cells. These findings suggest that PHF19-207 holds potential as a diagnostic biomarker. In addition, in silico analyses indicate that this transcript may function as a long non-coding RNA involved in the regulation of gene expression. Further functional investigations are required to elucidate its precise role in colon carcinogenesis.

## 1. Introduction

According to 2022 data (https://gco.iarc.who.int/en, accessed on 15 November 2024), colorectal cancer (CRC) ranks as the third most commonly diagnosed cancer in both men and women, and is the second leading cause of cancer-related deaths globally. According to the World Health Organization (WHO), of all colorectal cancer cases, 70% are localized in the colon.

One of the hallmarks of cancer, including colon cancer, is its dynamic transcriptional landscape and usage of alternative promoters. Expression of many human protein-coding genes is regulated by alternative promoters. Recent findings from an extensive pan-cancer transcriptome analysis revealed differential expression of two alternative *PHF19* gene promoters in malignant versus non-malignant gut mucosa [1].

The promoter upregulated in colon and rectal cancer gives rise to the PHF19-207 transcript, suggesting a potential tumor-promoting function. The *PHF19* gene encodes PHD finger protein 19, a component of the polycomb repressive complex 2 (PRC2), which is involved in H3K27 methylation, a chromatin modification linked to transcriptional repression [2]. The target genes of *PHF19* protein are implicated in processes such as proliferation, differentiation, angiogenesis, and the organization of the extracellular matrix [3]. The role of *PHF19* protein in malignant transformation has been demonstrated in several malignancies, with its tumor-promoting role in colorectal cancer revealed only recently [4,5].

The *PHF19* gene is located at 9q33.2 and encompasses 39245bp. A set of 14 transcripts was identified from this gene, with major transcript PHF19-202 (ENST00000373896) encoding a 580-amino-acid-long protein. According to the Ensembl database, the majority of other transcripts are either truncated or have an undefined coding sequence. Elements of the non-coding transcriptome are increasingly recognized as key contributors to the complexity of the genome; however, their specific roles remain largely unexplored.

The objective of our study was to examine the expression of PHF19-207 in colon cancer, assess its potential as an early biomarker for colorectal cancer, and evaluate its functional implications using in silico tools, as the function of this transcript has not been previously characterised.

## 2. Materials and Methods

### 2.1. In Silico Analysis of the PHF19 Gene Promoters

The promoter sequences of the *PHF19* gene were defined as 1kb regions both upstream and downstream of the two transcription start sites (TSSs) identified as differentially active in colorectal cancer [1]. These promoter sequences were retrieved in FASTA format from the human GRCh38.p13 assembly using the Ensembl genome browser. To analyze characteristic motifs within these sequences, the Motif Finder tool of the Integrative Genomics Viewer (IGV) program was employed [6]. The distribution of GC boxes was examined using the MethPrimer 2.0 tool (http://www.urogene.org/methprimer/) [7].

Four available bioinformatic tools were utilized to predict the presence of consensus sequences for potential transcriptional regulator binding within the *PHF19* gene promoter: Alggen PROMO 2.0 (https://alggen.lsi.upc.es, https://bio.tools/alggen), AliBaba 2.1 (https://gene-regulation.com/pub/programs/alibaba2/), CiiDER (https://ciider.com, https://bio.tools/CiiiDER), and TFBIND (https://tfbind.hgc.jp) [8,9,10]. Each of these four tools employs different algorithms, and their combined use enhances prediction robustness and allows for cross-validation results. Default query parameters and human libraries were applied in these analyses to ensure optimal performance of the mentioned tools and reproducibility. Only the positive results obtained from at least two algorithms for each transcriptional regulator were considered. The expression levels of the identified regulators in colon cancer and normal gut mucosa were analyzed using the Gene Expression Profiling Interactive Analysis 2 (GEPIA) tool (http://gepia.cancer-pku.cn/).

The list of genetic variants in the PHF19 gene promoter sequences was extracted from the Ensembl database (global MAF:0.005-0.5, class: SNP, clinical consequences: all, consequences: all) to map variants occurring in the predicted binding sites of transcriptional regulators.

### 2.2. In Silico Analysis of PHF19-207

The sequence of the PHF19-207 transcript was retrieved as a FASTA file using the human GRCh38.p13 assembly from the Ensembl genome browser (ENST00000456291).

The coding potential of the transcript was assessed using the LCG Coding Potential Prediction 2.0 tool (https://ngdc.cncb.ac.cn/lgc/) and Coding Potential Calculator 2 (http://cpc2.gao-lab.org/), while the prediction of its secondary structure was conducted with the RNAfold tool. To evaluate the expression of PHF19-207 in normal and tumor colon tissues, the AnnoLnc2 tool was utilized, based on publicly available expression data. Additionally, the transcript’s cellular localization was predicted using AnnoLnc2, with further validation performed using the lncLocator 1.0 (http://www.csbio.sjtu.edu.cn/bioinf/lncLocator/) and lncLocator2 (http://www.csbio.sjtu.edu.cn/bioinf/lncLocator2/) tools [11].

Homologue miRNA sequences were extracted using miRbase 22.1 (http://mirbase.org) and the mirDB 6.0 custom prediction tool (http://mirdb.org) [12,13]. Potential interactions between detected miRNAs and PHF19-207 were predicted using RNA22 v2 tool (http://cm.jefferson.edu/rna22/Interactive) and BiBiServ2 RNAhybrid 2.1.2 tool (http://bibiserv.cebitec.uni-bielefeld.de/rnahybrid, https://bio.tools/rnahybrid) [14,15].

### 2.3. Cell Cultures

The cell lines utilized in this study were derived from human colon tissue, including the immortalized colonic epithelial cell line HCEC-1CT (CVCL_AQ45) isolated from healthy tissue (Evercyte GmbH, Wien, Austria) and a panel of colon cancer cell lines: HCT 116 (CVCL_0291), HT-29 (CVCL_0320), CaCo-2 (CVCL_0025), SW480 (CVCL_0546), DLD-1 (CVCL_0248), and SW620 (CVCL_0547) (ATCC, Manassas, VA, USA). All cell lines were cultured in Dulbecco’s Modified Eagle Medium (DMEM; Capricorn Scientific, Ebsdorfergrund, Germany) supplemented with 10% fetal bovine serum (FBS; Capricorn Scientific, Germany) and 1% antibiotic/antimycotic solution (Capricorn Scientific, Ebsdorfergrund, Germany) in a 5% CO_2_ atmosphere at 37 °C. Cells were subcultured once they reached 70–80% confluence using 1× trypsin/EDTA (Capricorn Scientific, Ebsdorfergrund, Germany). To ensure biological relevance, cells were cultivated in triplicate. All cell lines were confirmed to be free from mycoplasma contamination.

Non-malignant (HCEC-1CT) and malignant cell lines representing different tumor stages according to the Dukes’ classification (HCT116, DLD-1, and SW620) were cultured in 3D as spheroids. To generate the spheroids, adherent cells were detached using 1× trypsin/EDTA (Capricorn Scientific, Ebsdorfergrund, Germany) and counted with a standard hemocytometer. Approximately 2 × 10^5^ cells per well were seeded in a 24-well Nunclon™ Sphera™ Dish (Thermo Fisher Scientific, Waltham, MA, USA), designed for low cell attachment, containing 1 mL of the complete culture medium described earlier. Spheroids were cultured for 7 days in a humidified incubator at 37 °C with 5% CO_2_. To maintain nutrient levels and remove dead cells, media changes were performed every 2–3 days. During incubation, spheroids were monitored daily under a phase-contrast microscope for shape, growth and compactness. Compact spheroids were defined based on the following morphological criteria: spherical morphology with clearly defined borders, absence of fragmented edges or loosely attached cells, and uniform in size distribution within biological replicates. Compact spheroids were collected under a microscope to ensure that only live cells, free from debris, were selected for subsequent total RNA extraction.

### 2.4. Cell Transfection

HCEC-1CT cells were seeded at a density of 2 × 10^5^ and cultured for 24h in DMEM medium without antibiotic/antimycotic solution. Cells were counted using a standard hemocytometer. Transient transfection with the EGFP-KRAS-G12V plasmid (#164925, Addgene, Watertown, MA, USA) and control plasmid was performed using LipofectamineTM 3000 (Thermo Fisher Scientific, Waltham, MA, USA) according to the manufacturer’s protocol. Transfection was performed in triplicate. KRAS expression was confirmed by the presence of GFP fluorescence, and cells were collected after 24 h.

### 2.5. RNA Extraction

Total RNA was extracted from approximately 8 × 10^6^ adherent cells (2D cultured cells) and spheroids (3D cultured cells) from two 24-well plates using the PureLink™ RNA Mini Kit (Thermo Fisher Scientific, Waltham, MA, USA) following the manufacturer’s protocol. RNA from transfected cells was isolated using the same procedure. For RNA isolation from the HCEC-1CT and SW620 cell compartments, the Cytoplasmic & Nuclear RNA Purification Kit (Norgen Biotek Corp., Thorold, ON, Canada) and the Cell Culture Media Exosome Purification and RNA Isolation Midi Kit (Norgen Biotek Corp., Thorold, ON, Canada) were employed, adhering to the respective manufacturer’s protocols. The concentration and purity of the extracted RNA were assessed by measuring absorbance at 260 nm and 280 nm using a BioSpec-nano spectrophotometer (Shimadzu, Kyoto, Japan).

### 2.6. RNA Sequencing

High-throughput next-generation RNA sequencing was conducted by Novogene (UK) Company Limited (Cambridge, UK). Total RNA from the cultivated spheroids underwent quality control (QC), which included 1% agarose gel electrophoresis, Nanodrop spectrophotometry to assess RNA concentration and purity, and Agilent 2100 analysis to evaluate the RNA Integrity Number. Library preparation involved ribosomal RNA depletion, facilitating RNA enrichment for gene expression profiling of both coding and non-coding transcripts. Sequencing was performed using the Illumina NovaSeq6000 platform, generating paired-end 150 bp reads. Bioinformatics analysis comprised quality control, mapping of the reads to the GRCh38 human reference genome, and quantification of gene expression levels using Novogene’s established pipeline, which provided raw counts. A Sashimi plot of the Binary Alignment Map (BAM) files was generated using the Integrative Genomics Viewer (IGV).

### 2.7. Quantitative Real-Time PCR (qRT-PCR)

Reverse transcription of total RNA, isolated from both 2D and 3D cultured cells (2 μg) and cell compartments (0.1 μg), into complementary DNA (cDNA) was carried out using the High-Capacity cDNA Reverse Transcription Kit (Applied Biosystems, Waltham, MA, USA), following the manufacturer’s protocol. The reaction conditions included 10 min at 25 °C, 120 min at 37 °C, and 5 min at 85 °C.

Relative expression of PHF19-207 was quantified in triplicate using quantitative real-time PCR (qRT-PCR) with Power SYBR Green PCR Master Mix (Thermo Fisher Scientific, Waltham, MA, USA). The specificity of the amplification products was confirmed by performing a melting curve analysis. Glyceraldehyde-3-phosphate dehydrogenase (GAPDH) served as the endogenous control for all measurements. The forward primer was designed to bind to the retained intron of the PHF19-207 transcript, and the reverse primer targeted the retained intron–exon junction to ensure reaction specificity. The sequences of the primers used for relative quantification are provided in Table 1.

qRT-PCR was conducted using the 7500 Real-Time PCR System (Applied Biosystems, Waltham, MA, USA), and relative quantification was calculated using the 2-dCt method. The reaction conditions consisted of 2 min at 50 °C, 10 min at 95 °C, followed by 40 cycles of 15 s at 95 °C and 1 min at 60 °C. The expression levels of PHF19-207 were determined and normalized to the endogenous control.

### 2.8. Data Used in the Study

Publicly available high-throughput RNA sequencing data (GSE152562, GSE164541, and GSE254832) were obtained from the National Center for Biotechnology Information’s Gene Expression Omnibus database, NCBI GEO (https://www.ncbi.nlm.nih.gov/geo/) [16,17,18]. The corresponding raw sequencing files in FASTQ format were downloaded from the Sequence Read Archive (SRA) (https://www.ncbi.nlm.nih.gov/sra) under accessions SRP267478, SRP301216, and SRP487433, respectively. Downloads were facilitated using the SRA Explorer web application (https://sra-explorer.info/). The sequencing reads were aligned to the GRCh38 human reference genome, and the expression levels of *PHF19* transcripts were quantified using the HISAT2 and StringTie tools [19]. The expression of PHF19-207 in clinical samples was assessed using the UCSC Xena Browser web server (https://xenabrowser.net/), comparing The Cancer Genome Atlas (TCGA) colon adenocarcinoma and Genotype-Tissue Expression (GTEx) colon datasets, with data accessed on 15 November 2024 [11]. Additionally, PHF19-207 expression was evaluated across other solid tumor types using TCGA data available through the UCSC Xena Browser web server.

### 2.9. Statistical Analysis

Statistical analysis was conducted using GraphPad Prism v9 software. Data are presented as percentages and as the mean ± standard deviation. The distribution of the data was assessed using the Shapiro–Wilk test. Differences between groups were analyzed using the independent samples *t*-test and analysis of variance (ANOVA), followed by Dunnett’s post hoc test. A *p*-value of ≤0.05 was considered statistically significant.

## 3. Results

### 3.1. In Silico Analysis of the PHF19 Gene Promoters

**Figure 1:**
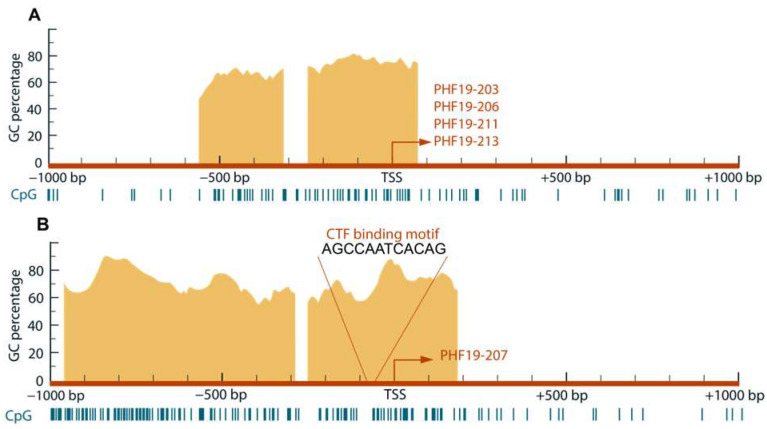
Schematic representation of analyzed promoter sequences with positions relative to TSS of predicted CpG islands and CTF/NFI transcription factors binding site. (**A**) Downregulated promoter sequence showing two CpG islands. The common transcription start site (TSS) of four transcripts is shown within one CpG island. (**B**) Upregulated promoter shows two CpG islands and a CTF/NFI binding site near TSS, giving rise to the PHF19-207 transcript.

The analysis of characteristic elements revealed an atypical structure in both analyzed PHF19 gene promoters, located at the genomic positions chr9:120867881-120869881(-1) for the downregulated promoter and chr9:120875433-120877433(-1) for the upregulated promoter. Both promoters were found to be TATA-less, with a variable number of CCAAT and GC boxes. The presence of CpG islands was predicted in both promoters (Figure 1).

Prediction of transcription regulator binding motifs identified the differential presence of the CTF/NFI binding motif in *PHF19* gene promoters. A protein family, CTF/NFI, containing a member with dual roles in cell growth, is predicted to have a binding motif in the promoter upregulated in cancer. According to GEPIA, the CTF transcription factor shows lower expression in tumor tissue, with a log2FC value of −2.209.

Of the genetic variants mapped in the promoter regions (9 in the first and 13 in the second promoter), no variants were present in the regions where the transcriptional regulator binding was predicted.

### 3.2. In Silico Analysis of the PHF19 Gene Transcripts

**Figure 2:**
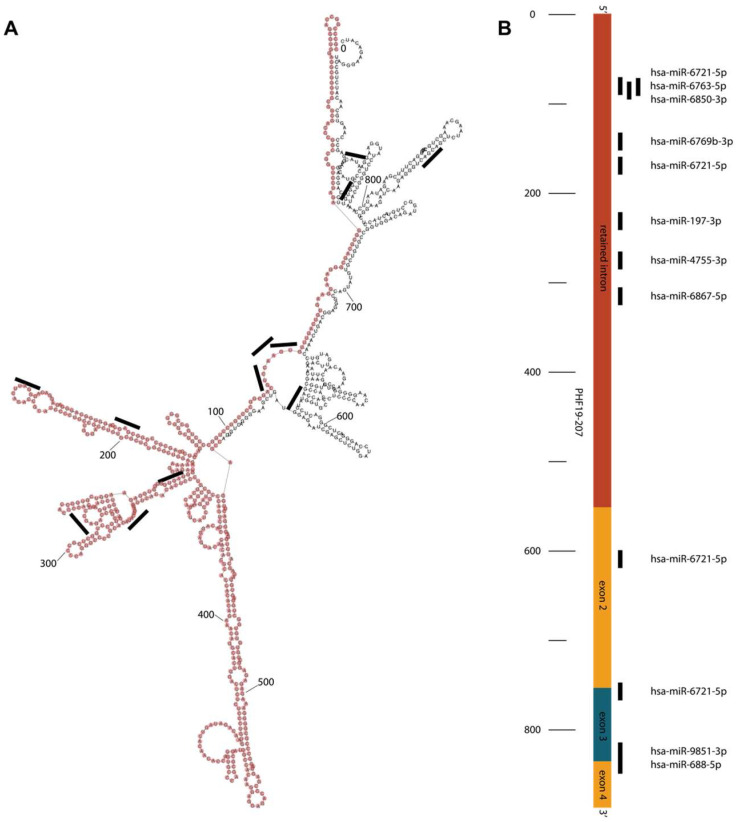
(**A**) Predicted secondary structure of the PHF19-207. The retained intron of the PHF19-207 transcript is highlighted with pink dots. The miRNA binding positions are illustrated with black lines. (**B**) Schematic representation of the microRNA predicted binding sites in the PHF19-207 transcript primary sequence.

The PHF19-207 transcript, produced by the promoter upregulated in cancer, was examined using various in silico tools to assess its potential function. Based on the Coding Potential Calculator and LCG Coding Potential Prediction tools, PHF19-207 was classified as non-coding. The RNAfold tool predicted the secondary structure of the PHF19-207 transcript with no repeated elements (Figure 2A). The AnnoLnc2 and lncLocator2 indicated nuclear localization of the PHF19-207 transcript, while the lncLocator predicted its localization mainly in exosomes. The RNA22 tool, used for the prediction of interactions with miRNA, identified several miRNAs that could bind to PHF19-207, mostly within the retained intron (Figure 2).

### 3.3. Quantification of PHF19 Gene Transcripts by RNA Sequencing in Colon Cell Lines Cultivated in 3D

RNA sequencing identified 10 PHF19 transcripts, each detected in at least one of the analyzed cell lines, with four transcripts deemed non-expressed based on the FPKM threshold of 0.3 (Table 2) [20].

**Figure 3:**
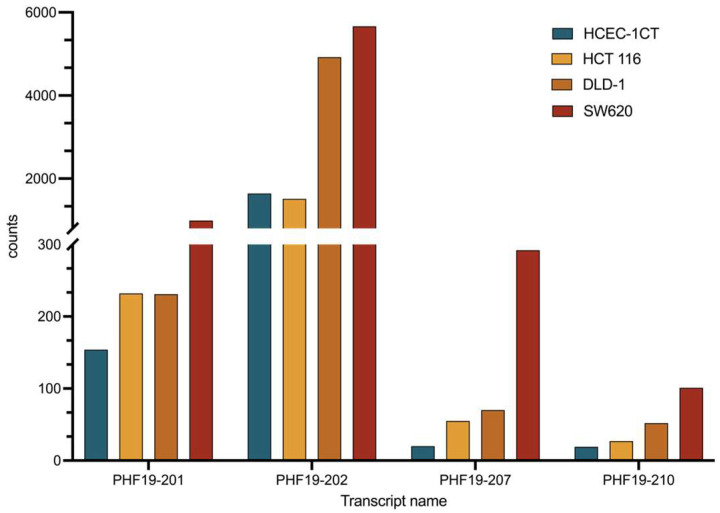
Expression of the *PHF19* transcripts that exhibit a pattern of increased expression in colon cancer cell lines (early-stage colon cancer HCT 116 and late-stage colon cancer DLD-1 and SW620) compared with the non-malignant HCEC-1CT colon cell line, cultivated in 3D, measured by RNA sequencing.

**Figure 4:**
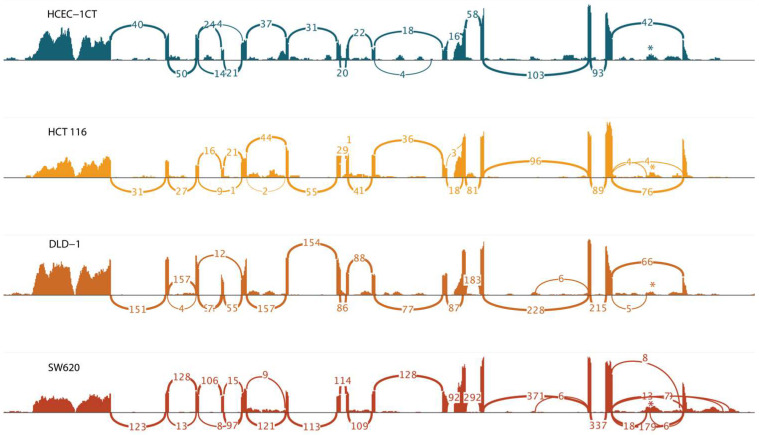
Sashimi plot of the *PHF19* gene region showing increased utilization of retained intron sequence of the PHF19-207 transcript in colon cancer cell lines. The retained intron of the PHF19-207 is highlighted with *.

The majority of the transcripts were present at low quantities (<1 FPKM). Transcripts PHF19-201 and PHF19-202 were moderately expressed in all analyzed cell lines (FPKM between 1 and 10). Their expression was increased in cell lines representing late-stage colon cancer (DLD-1 and SW620), but their expression in the cell line representing early-stage colon cancer (HCT 116) was similar to that of a non-malignant cell line (Figure 3). The other two transcripts that showed an increase in expression in colon cell lines were PHF19-207 and PHF19-210. They were elevated in all colon cancer cells, with the elevation more prominent towards the late stage of the disease. RNA sequencing also illustrated differential splicing events in sequenced cell lines (Figure 4).

**Figure 5:**
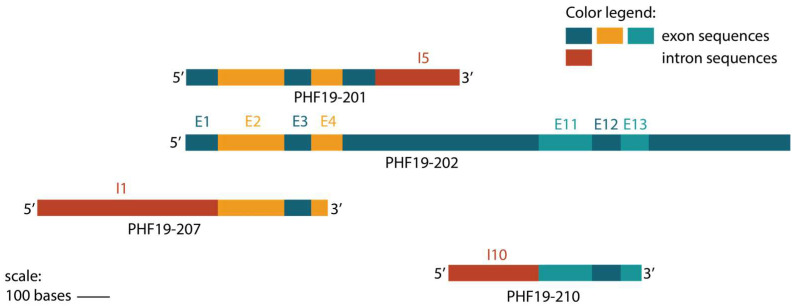
The sequence structure of transcripts with an observed increased expression during colon cancer stages, with their shared exons (E) and retained introns (I). The transcripts are aligned with the main PHF19-202 transcript.

A comparison of upregulated transcripts showed an alignment in coding sequences between PHF19-207, PHF19-201 and exons 2, 3, and 4 of the referent PHF19-202 is shown in Figure 5. However, transcript PHF19-210 has a coding sequence containing exons 11, 12, and 13 (Figure 5).

### 3.4. Analysis of Publicly Available Sequencing Data

**Figure 6:**
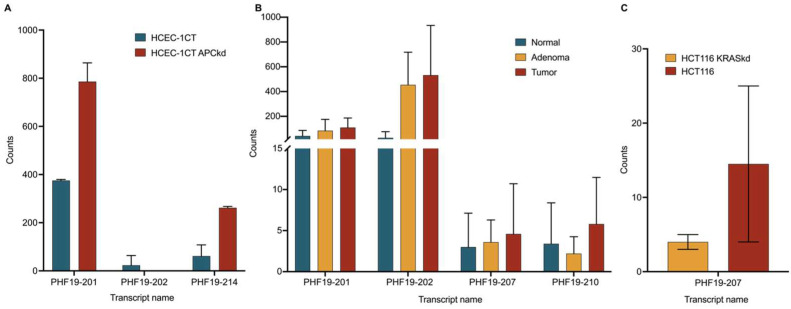
Expression of PHF19 transcripts measured with RNA sequencing. (**A**) PHF19 transcripts expressed in HCEC-1CT and HCEC-1CT APC knockdown cells (GSE152562), (**B**) PHF19 transcripts in normal, adenoma and tumor tissue (GSE164541), and (**C**) HCT116 and HCT116 KRAS knockdown cells (GSE254832).

GSE152562 is the dataset consisting of HCEC-1CT and HCEC-1CT APC knockdown sequencing triplicates. An analysis of the GSE152562 dataset revealed no expression of the PHF19-207 transcript, either before or after knockdown of the APC gene (Figure 6A). However, an analysis of the expression of this transcript in mucosa, adenoma, and tumor colon tissues (dataset GSE164541) showed a prominent increase in expression of PHF19-207 (Figure 6B). This dataset also showed moderate upregulation of PHF19-201 and PHF19-202 in adenoma and tumor tissue in comparison to mucosa. However, no trend was observed in the expression of PHF19-210 among healthy mucosa, adenoma and tumor tissues. GSE254832 is the dataset consisting of RNA sequencing data of the HCT 116 colorectal cancer cell line and its KRAS knockdown transfectant. The HCT 116 cell line and HCT 116 KRAS knockdown transfectant showed different but not statistically significant changes in the expression of PHF19-207 transcript (Figure 6C). The UCSC Xena Browser web server indicated down-regulation of PHF19-207 in GTEx normal colon tissue and its upregulation in TCGA colon cancer tissue samples, with a *p*-value of 1.442 × 10^−9^. According to an evaluation of PHF19-207 expression in other types of solid tumors from TCGA data, this transcript is mostly expressed in colon and rectum tumor tissues with mean FPKM values of 0.371 and 0.351, respectively. The TCGA data from cancer tissues originating from the cervix, esophagus, stomach, thymus, testis, lung, brain, skin, bile duct, and pancreas exhibit lower mean FPKM values, ranging from 0.2 to 0.1.

### 3.5. Expression Analysis of PHF19-207 in Colon Cell Lines Cultivated in 2D and 3D by qPCR

**Figure 7:**
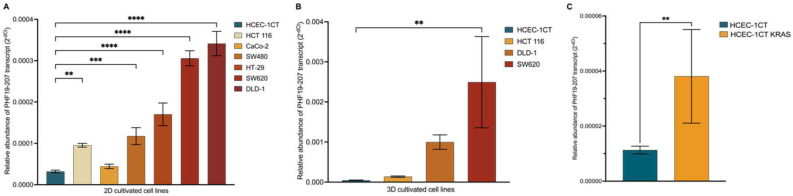
Expression of PHF19-207 in colon cell lines cultivated in 2D (**A**), 3D (**B**), and HCEC-1CT cells with overexpressed KRAS (**C**) measured by qPCR. Statistical significance is shown with symbols: **—*p* ≤ 0.01, ***—*p* ≤ 0.001, ****—*p* ≤ 0.0001.

The relative abundance of the PHF19-207 transcript was analyzed in both human non-malignant and malignant colon cell lines cultured in 2D and 3D. In the 2D-cultured colon cell lines, the expression of PHF19-207 was relatively low in the non-malignant HCEC-1CT cell line, while it was elevated in all malignant cell lines (Figure 7A,B). Notably, the increase in PHF19-207 expression was more pronounced in cell lines representing advanced stages of colon cancer. In 3D-cultured colon cell lines, RNA sequencing results confirmed this trend, with malignant cell lines exhibiting higher expression levels compared to non-malignant cell lines. The relative abundance of PHF19-207 in HCEC-1CT cells with overexpressed KRAS showed significant expression of PHF19-207 compared to HCEC-1CT cells (*p* = 0.0032) (Figure 7C). Transcript PHF19-207 was detected in the nucleus of both HCEC-1CT and SW620, and the exosomes of SW620.

## 4. Discussion

Alterations in alternative transcription initiation have been observed in various pathologies, including cancer [21,22]. The diagnostic and prognostic potential of alternative promoters and transcripts has been established in several cancers, including colorectal cancer, multiple myeloma, prostate cancer, and hepatocellular carcinoma [23,24,25,26]. This study was conducted using cell lines, high-throughput sequencing and computational tools to evaluate the potential of the transcript PHF19-207 as a biomarker for early colon cancer and to explore its possible role in tumor promotion. The hypothesis on its involvement in the early stages of colon cancer was derived from a previous comprehensive study that had screened for the deregulation in the genes’ promoter activity between tumor and non-tumor tissue and found deregulation in the activity of the PHF19 gene promoters [1]. The promoter down-regulated in colon cancer tissue was found to be upregulated in glioblastoma. The other promoter was found to be upregulated in colon and rectal cancer, kidney cancer, stomach cancer, and chronic lymphocytic leukemia.

The presence of characteristic motifs was similar in the two analysed promoters of the PHF19 gene. According to in silico predictions, both promoters are located within CpG islands. A previous study investigated transcriptional activity of gene promoters in colon cancer using the H3K4me3 mark [4]. Results of that study overlap with findings that the upregulated promoter is located in a transcriptionally active region of the PHF19 gene.

The potential binding of transcriptional regulators to the promoter sequences was assessed using four distinct bioinformatics tools. Regulators predicted by at least two of these tools to bind to either of the promoters were considered for further analysis. Based on the presence of their binding motifs in the promoter sequences, the CTF family was predicted to bind to the promoters upregulated in colon cancer. Data from GEPIA showed that the expression level of CTF is lower in colon cancer compared to the healthy gut mucosa. Lower expression of CTF proteins in combination with upregulation of the second promoter in colon cancer can be explained by their dual activity, since the family includes both activators and repressors. Since NFI/CTF transcription factors have both oncogenic and tumor suppressor potential, depending on the type of carcinoma, their role in regulating PHF19 gene promoters should be further investigated [27].

Transcript PHF19-207 is 888 nucleotides long and classified as protein-coding according to Ensembl. Its computationally mapped protein isoform consists of 106 amino acids. In silico evaluation of PHF19-207 suggests that this RNA may be non-coding rather than coding. It has low coding probability according to the LCG Coding Potential Prediction tool and Coding Potential Calculator tool, and the AnnoLnc2 tool indicates its localization in the nucleus. Although the localization data are not available for colon cell lines, they are consistent for a variety of other tissues, indicating that this transcript is predominantly retained in the nucleus regardless of the tissue. Another bioinformatic tool, lncLocator, predicts the localization of this transcript in exosomes. In silico data also point to the upregulation of PHF19-207 in colon cancer tissue samples in comparison to normal colon mucosa. Overall, in silico data indicate that PHF19-207 may be a long non-coding RNA involved in gene regulation and/or signalling. Additionally, its role in communication between colon cancer cells and the tumor microenvironment can include the RNA fluorescence in situ hybridization method.

In silico data also predict binding of nine microRNA molecules for the transcript PHF19-207. These microRNAs are predicted to bind mainly towards 5′ (within intron 1) and 3′ ends of the transcript, and only a couple of them have overlapping binding sites. Most of the miRNAs bind to the predicted loops of the RNA secondary structure. For some of the microRNA molecules predicted to bind to PHF19-207, anti-tumor roles were demonstrated, while others are not yet characterized [28,29]. These data suggest that the retained intron of the PHF19-207 transcript may act as a microRNA sponge, which is in line with its proposed tumor-promoting role in colon tumorigenesis. MicroRNA hsa-6721-5p has four binding sites in the PHF19-207 sequence. Simultaneous binding of multiple miR-6721-5p may modulate transcript secondary structure and stability, suggesting a possible anti-tumor role of this miRNA. As such, the retained intron of the PHF19-207 transcript could be involved in the regulation of colon carcinogenesis, and further study of functional properties is required for understanding its role.

However, according to the Ensembl database, this transcript is protein-coding. The translated protein of this transcript is suggested to be 106 amino acids long. In comparison with the reference transcript, which transcribes a protein with 580 amino acids, this protein might have different roles in cells. Recently, studies suggested the existence of small open reading frames (sORF) on long non-coding RNA molecules that are engaged by ribosomes [30]. Micropeptides originating from these ORFs deviate from canonical peptide sequences and are, on average, about 100 amino acids long. PHF19-207 predicted peptide fits this description. The hypothesis that PHF19-207 is a protein-coding long non-coding RNA should be further investigated. Confirmation of this hypothesis would require analysis of ribosome recruiting on this transcript, mass spectrometry, in vivo translation, and custom-made antibodies for Western blot [31].

The results of the public data showed no expression of PHF19-207 in either the wild-type or APC knockdown HCEC-1CT cell line [16]. It can be assumed that this transcript is not the result of a first-ever genetic alteration in the canonical colorectal carcinogenesis pathway. However, we have to consider that the technique used in that study, Illumina NextSeq 500, does not have the same sequencing depth as the NovaSeq 6000 used in our study. This may explain why we quantified the lowly expressed PHF19-207 transcript in the HCEC-1CT cell line and observed upregulation in cell lines representing other stages of tumor development.

Results of public sequencing data also showed no statistically significant changes in PHF19-207 expression between HCT 116 cell lines with wild-type and KRAS knockdown [18]. Within that study, RNA of wild-type and transfected cell lines was sequenced in duplicates. Considering that, we overexpressed GFP-labeled KRAS G12V mutant peptide in the normal colon mucosa cell line HCEC-1CT. Results showed statistically significant changes in PHF19-207 expression in cell lines with overexpressed, mutated KRAS versus without overexpressed KRAS. This experiment suggests that the KRAS mutation could be one of the first genetic deregulations that drive upregulation of the PHF19-207 transcript.

Analysis of publicly available sequencing data showed a slight increase in the expression of PHF19-207 in the tumor in comparison to normal tissue, but without statistical significance [17]. The study that produced this public data analyzed triplicate tissue samples from five patients with colorectal cancer. A comparison between TCGA and GTEx data suggested that there is a difference between tumor and normal gut mucosa. However, this data did not include adenomas and showed a clear difference in tumor staging. We suggest further research on the expression of this transcript in clinical samples in larger patient groups with tumors in different stages and more sensitive methods, such as ddPCR.

The PHF19-201 transcript is shown to be significantly expressed in the HCEC-1CT APC knockdown cell line. Our data showed similar expression between HCEC-1CT and the HCT 116 cell line. However, its expression in DLD-1 and SW620 was significantly upregulated. Considering that the HCT 116 cell line has a functional APC gene, we can conclude that this transcript could be a marker of APC deregulation in colon cancer.

The results of the transcriptional profiling confirm biomarker potential and are also in line with the proposed tumor-promoting role of the PHF19-207 transcript. The expression analysis of cells cultured in 2D, conducted using qPCR, revealed that PHF19-207 expression was elevated in all malignant cell lines compared to the non-malignant HCEC-1CT cell line (by 2 to 5-fold). A more significant increase in expression was observed in the cell lines derived from advanced stages of colon tumors (HCT 116 Dukes’ A category; HT-29, CaCo-2 and SW480 Dukes’ B category; DLD-1 and SW620 Dukes’ C category). Similar results were obtained when the expression of PHF19 gene transcripts was analysed in cells cultivated in 3D using RNA sequencing, where PHF19-207 expression was elevated 2 to 7.5-fold in the malignant cells vs. the non-malignant cell line. Also, aberrant splicing events are occurring more in cells representing later stages of colon cancer. Cell lines were cultured in 3D to ensure that the resulting transcriptomes accurately represent those of cells in their native environment. PHF19-207 was detected in the nucleus of both HCEC-1CT and SW620 cell lines, and SW620 exosomes using qPCR, which also validated the in silico prediction results of transcript localization. Exosomes are one of the regulators of cell-to-cell communication [32]. Their role in cancer development and aggressiveness is demonstrated in breast cancer [33]. Increased secretion of PHF19-207 via exosomes in colon cancer could elucidate its mechanism of action in cancer development and should be further explored.

Transcript PHF19-210 showed a similar expression pattern as PHF19-207 in expression data from cell lines, and considering its undefined coding sequence and length (588 nucleotides), it may also be considered by future studies as non-coding RNA with a potential role in tumorigenesis. Its expression from clinical NGS data does not suggest it could be used as a biomarker.

The results of this study provide us with detailed data on PHF19 expression through the development of colon cancer and suggest the potential use of PHF19-207 as a biomarker of early colon cancer. However, several limitations should be acknowledged. First, the findings of this study rely primarily on data from established cell lines and publicly available RNA sequencing data, which may not fully capture the complexity or heterogeneity of primary tumor tissues. Additionally, while differential expression and splicing patterns of PHF19-207 are clearly demonstrated, functional validation experiments are required, and, therefore, the biological role of this isoform remains speculative. Although PHF19-207 contains a retained intron, its consistent and elevated expression across samples suggests that it is not subject to effective nonsense-mediated decay (NMD), supporting the potential functional relevance of this isoform. Previous studies illustrate that non-coding transcripts originating from loci of protein-coding genes could have roles in different molecular processes in a malignant cell [34]. High-risk patients undergoing screening for colorectal cancer could benefit the most from the implementation of early colon cancer biomarkers, such as PHF19-207. With further functional characterization and description of transcript behavior in tumor cells under therapeutics, we could estimate the different aspects of biomarker potential.

## 5. Conclusions

This study has demonstrated the potential of the transcript PHF19-207 for early colon cancer detection and proposes a dual role of PHF19-207 that should be further investigated. Its retained intron could function as an miRNA sponge, and interaction partners of this transcript should therefore be analyzed. The presence of the sORF in its sequence suggests the potential existence of a micropeptide, which requires experimental validation. The consistent differential expression between malignant vs. non-malignant cell lines and tumor vs. normal tissue samples confirms its biomarker potential. Further studies should aim to investigate the functional relevance of the PHF19-207 transcript in colorectal cancer.

## Figures and Tables

**Table 1:** The primer sequences used for qRT-PCR.

Target	Forward Primer	Reverse Primer
PHF19-207	5′-GATAGTCACAACACCAGGTGCC-3′	5′-CTTCCCCTGACACTGGCTCC-3′
GAPDH	5′-GTGAAGGTCGGAGTCAACG-3′	5′-TGAGGTCAATGAAGGGGTC-3′

**Table 2:** IDs and names (Ensembl GRCh38.p13 assembly) of the identified transcripts using the RNA sequencing of the 3D cultivated cell lines.

Transcript ID	Transcript Name
ENST00000312189	PHF19-201
ENST00000373896	PHF19-202
ENST00000436309	PHF19-204
ENST00000439674	PHF19-205
ENST00000456291	PHF19-207
ENST00000462229	PHF19-208
ENST00000464712	PHF19-209
ENST00000467266	PHF19-210
ENST00000474402	PHF19-211
ENST00000487555	PHF19-213

## Data Availability

The dataset is available upon request from the authors.

## Abbreviations

The following abbreviations are used in this manuscript:

PHF19	PHD finger protein 19
CRC	Colorectal cancer
PRC2	Polycomb repressive complex 2
TSS	Transcription start site
IGV	Integrative Genomics Viewer
miRNA	MicroRNA molecule
DMEM	Dulbecco′s Modified Medium
FBS	Fetal bovine serum
bp	Base pairs
GAPDH	Glyceraldehyde-3-phosphate dehydrogenase
NCBI GEO	National Center for Biotechnology Information’s Gene Expression Omnibus
ANOVA	Analysis of variance
NFI/CTF	Nuclear Factor I/CAAT box transcription factor
sORF	Small open reading frame
